# Ideology in organizational cognitive neuroscience studies and other misleading claims

**DOI:** 10.3389/fnhum.2013.00834

**Published:** 2014-01-16

**Authors:** Dirk Lindebaum

**Affiliations:** The University of Liverpool Management SchoolLiverpool, UK

**Keywords:** ideology, neuroscience, organizational behavior, organizational cognitive neuroscience, organization science

As part of this forum on “*Society, Organizations and the Brain*,” Butler (2014) contributed an article on how to operationalize interdisciplinary research by way of introducing “a model of co-production in organizational cognitive neuroscience (OCN)” (p. 1).

While I appreciate his work as an extension of prior research, there are some misleading claims in his article in terms of associating my previous work with what he terms “science ideology” (a term he does not define), and a misleading representation of key arguments presented in that body of work (Lindebaum, 2013b). Consequently, my aim in this article is twofold. First, I demonstrate that Butler uses the term “ideology” incorrectly. Second, I contrast his depiction of my work with what it actually states. Note that, consistent with previous work (Lindebaum, 2013a), I am explicit that a multitude of opinions on this seemingly touchy topic is likely to yield richer insights than any one dominant view alone. However, I highlight a need for accurate usage of terms and accurate engagement with each others' work, however much we might beg to differ on the topic.

## Ideology in science

The topic of ideology has been a contested line of inquiry in management studies for some time (see e.g., Alvesson and Willmott, 1992; Raftopoulou and Hogg, 2010). Key to Butler's(2014) brief exegesis on ideology is the role of dominant actors when knowledge becomes “ideological and biased in favor of particular actors through a conflictural process” (p. 4). However, more elaboration is in order on a topic as complex as ideology. To begin with, it is important to understand what scholars mean when they refer to ideology. For instance, Van Dijk (1995) defines ideology along these lines.

“Ideologies are basic frameworks of social cognition, shared by members of social groups, constituted by relevant selections of socio-cultural values, and organised by an ideological schema that represents the self-definition of a group. Besides their social function of sustaining the interests of groups, ideologies have the cognitive function of organizing the social representations (attitudes, knowledge) of the group, and thus indirectly monitor the group-related social practices and hence also the text and talk of its members” (p. 248).

In other words, ideologies are characterized as a system of values, ideas, and beliefs that seek to legitimize extant hierarchies and power relations and preserve group identities. Therefore, ideology operates in the process of meaning in everyday life by way of common-sense and taken-for-granted assumptions that work to legitimize existing power relations (e.g., Fairclough, 1992). The focus upon meaning implies that ideology is viewed as an imaginary relationship of individuals to their real world, rather than a reflection of the real world (Althusser, 1971). If we take ideology and combine it with the scientific knowledge we share, it is clear that knowledge is *never free* of ideological influences. Thus, neither the work of advocates nor the work of skeptics of OCN is ideologically free. That is, neither camp can cast off its “ideological boundedness” (Fairclough, 1995). The problem arises if, among a set of ideologies, some exercise a more powerful influence than others, which then starts constrain some lines of enquiry while privileging others.

Having defined “ideology,” it is now possible to examine Butler's(2014) association of my previous work with “science ideology.” He states that “within the UK,” I am seemingly “a key voice for critique, however, [I am] perceived by colleagues as straying into science ideology” (p. 4). However, does this accurately reflect the power balance between advocates and skeptics of OCN? In terms of numbers of publications in flagship US management journals, my counting reveals a score of at least 15 to 0 in favor of advocates^1^, so I cannot see that my work is part of an existing hierarchy that dominates the field. In this respect, I am reminded of Gabriel's(2010) observation that “what gets published and what gets rejected … are barely concealed exercises in power and resistance … what gets published is one of the most political processes” in today's academia (p. 761). Thankfully, other thought-provocative and original journals like the *Journal of Management Inquiry* or *Human Relations* (Lindebaum and Zundel, 2013) have been more receptive to my work^2^.

I also would like to briefly reflect on Butler's(2014) words to the effect that I am “*perceived by colleagues* as straying into science ideology” (p. 4). The first part (in italics for emphasis) of that sentence requires attention. Specifically, I wonder whether Butler (2014) intended to make a factual statement, or whether his comment is based upon hearsay of the kind we can read in tabloids. If it is the former, the reader would appreciate evidence in support of his claim. If it is the latter, I am not sure whether this statement adds substance to his article.

## Misleading claims

The second point I would like to raise in response to Butler (2014) is his depiction of key points I offered previously. To explicate, consider the following first quote from his article:

“On the one hand, Lindebaum and Zundel (2013) rightly maintain that without explicit consideration of, and solutions to, the challenges of reductionism, the possibilities to advance leadership studies theoretically and empirically are limited” (p. 4).

While it is gratifying to see one's work being cited, it is also important that this is executed correctly in congruence with academic conventions of citation practice. In this case, the above statement is taken *ad verbatim* (starting with “maintain” and ending with “limited”) from Lindebaum and Zundel (2013) and, therefore, must be accompanied by the page number (i.e., p. 857). However, this is not the case.

There are two more problems with Butler's depiction of my work on the topic in the following statement:

“On the other hand, it has been argued that Lindebaum (2012) mischaracterizes neuro-feedback processes for the purpose of leader development, which then leads to misinformed statements about its potential ethics (Cropanzano and Becker, 2013)” (pp. 4–5).

The first problem is the reference to Lindebaum (2012). This study is not devoted in any way to OCN (instead it focuses on emotional standardizations at work). The second point pertains to the statement that I “*mischaracterize neuro-feedback processes*” as applied to leader development, “*which then leads to misinformed statements about its potential ethics.*” Readers who have perused my 2013(b) article will quickly see that I have characterized the neurofeedback process by first defining it according to the view of the *International Society for Neurofeedback and Research* (Hammond et al., 2011). I have also provided more characteristics of the neurofeedback process with reference to the Waldman et al. (2011) study (often using direct quotes from that study). Consequently, I cannot discern where a mischaracterization has occurred. The same applies to “*misinformed statements about potential ethics*,” a point allegedly made by Cropanzano and Becker (2013) in response to my article. What Cropanzano and Becker (2013) suggest, however, is that they “*strongly endorse* [my] *call for scholars and others to pay closer attention to … ethical concerns*” (p. 306) when neuroscience is used in leadership research. Of course, Cropanzano and Becker (2013) also offer divergent and complementary views on my critique, especially when they argue that my “*ethical inquiry does not go far enough*” and that “*a more complete analysis suggests that there are additional matters that should also be considered*” (p. 306). However, it is somewhat curious that Butler takes this to imply “*misinformed statements about its potential ethics*.” For further clarification on Cropanzano and Becker's(2013) article, please consult Lindebaum (2013a).

## Concluding thoughts

Butler (2014) deserves credit for bringing into the open the role of ideologies in the construction of knowledge, especially on a topic that enjoys hardly any substantive critique, least of all in flagship US management journals. However, the clarification of ideological charges against my work reveals that the exact opposite of Butler's (2014) argument is the case, namely, that advocates of OCN represent a dominant ideological movement, one which, through a system of ideas and beliefs, aims to legitimize extant hierarchies and power relations and preserve group identities as indicated by the score presented earlier. It is, therefore, important for future debates to be based upon informed views, which correctly and unequivocally reveal how the meaning of a term is employed. Since neuroscience as a theoretical and empirical toolkit is likely to further consolidate its influences in management studies (and how they fit with the theme of this research forum), it is all the more imperative to avoid terms being used to silence dissenting views or discredit prior work (for instance, by discarding them as lacking relevance and rigor). For a healthy unfolding of the debate, I suggest it is also necessary to engage more accurately with each others' work, for doing otherwise is likely to unnecessarily create deeper chasms rather than aiding to bridge them. I hope this article serves this purpose.

## Footnotes

^1^This score is calculated taking into account three publications in the *Journal of Management*, four in *Leadership Quarterly*, one in *Organization Science*, one in *Academy of Management Perspectives*, one in the *Journal of Applied Psychology*, two in *Strategic Management Journal*, and three in *Organizational Behavior and Human Decision Processes*. These publications represent those I am aware of, hence excluding any forthcoming or in press articles that have not been cited widely yet. I admit that there is a possibility that an article has escaped my attention. Even so, this is unlikely to fundamentally change the score presented. Due to space limitations, I cannot include the whole list here. However, it is available upon request.

^2^If we follow Duster (2006) in his claim that funding in the US is increasingly directed toward “markers inside the body” as predictors of socio-economic and health outcomes, then this tendency suggests another leverage of the OCN ideology and its associated power. The term “power” is most suitable here, as Scott (1992) defines it as having access to resources (in this case, research funding). Indeed, President Obama has just recently announced a US$100 million dollar brain-mapping research initiative. See http://blogs.nature.com/news/2013/04/obama-launches-ambitious-brain-map-project-with-100-million.html, accessed 21 October 2013.

## Note

This article by Lindebaum refers to a previous version of the opinion article “Operationalizing interdisciplinary research—a model of co-production in organizational cognitive neuroscience” by Butler, which first appeared online in provisional form on 11 October 2013 before undergoing final publication. In light of a potential conflict of interest identified after the initial peer review, the opinion article by Butler underwent an additional round of review and was then published in its current form. The final publication differs from the original version that first appeared online.
